# Thioredoxin‐interacting protein promotes activation and inflammation of monocytes with DNA demethylation in coronary artery disease

**DOI:** 10.1111/jcmm.15045

**Published:** 2020-02-10

**Authors:** Jialing Rong, Xianqun Xu, Yang Xiang, Guohua Yang, Xinliang Ming, Siying He, Bin Liang, Xiaokang Zhang, Fang Zheng

**Affiliations:** ^1^ Center for Gene Diagnosis Zhongnan Hospital of Wuhan University Wuhan China; ^2^ Demonstration Center for Experimental Basic Medicine Education of Wuhan University Wuhan China

**Keywords:** coronary artery disease, inflammation, methylation, monocytes, oxidative stress, thioredoxin‐interacting protein

## Abstract

Numerous studies have demonstrated that thioredoxin‐interacting protein (TXNIP) expression of peripheral blood leucocytes is increased in coronary artery disease (CAD). However, the molecular mechanism of this phenomenon remained unclear. DNA methylation plays important roles in the regulation of gene expression. Therefore, we speculated there might be a close association between the expression of TXNIP and methylation. In this study, we found that compared with controls, DNA methylation at cg19693031 was decreased in CAD, while mRNA expressions of TXNIP and inflammatory factors, NLRP3, IL‐1β, IL‐18, were increased. Methylation at cg19693031 was negatively associated with TXNIP expression in the cohort, THP‐1 and macrophages/foam cells. Furthermore, Transwell assay and co‐cultured adhesion assay were performed to investigate functions of TXNIP on the migration of THP‐1 or the adhesion of THP‐1 on the surface of endothelial cells, respectively. Notably, overexpressed TXNIP promoted the migration and adhesion of THP‐1 cells and expressions of NLRP3, IL‐18 and IL‐1β. Oppositely, knock‐down TXNIP inhibited the migration and adhesion of THP‐1 and expressions of NLRP3, IL‐18. In conclusion, increased TXNIP expression, related to cg19693031 demethylation orientates monocytes towards an inflammatory status through the NLRP3 inflammasome pathway involved in the development of CAD.

## 1 INTRODUCTION

Despite advances in prevention and treatment,^1^, ^2^ coronary artery disease (CAD) remains a threat to human health worldwide^3^, ^4^ due to atherosclerosis (AS) and its thrombotic complications. As we know, CAD is caused by combination of genetic and environmental factors.^3^ The traditional risk factors include smoking, diabetes mellitus, hypertension and hyperlipidemia. Besides, epigenetics, referring to the gene‐environment interaction, have attracted many attentions.^5^ Numerous evidences have demonstrated that DNA methylation, an important epigenetic modification, could regulate gene expression and involve in various biological and pathological processes, including CAD.^5^, ^6^ Our previous studies demonstrated that global DNA methylation level has certain significance in the pathogenesis of CAD.^7^ Because epigenetic processes are reversible, they are potentially valuable targets for disease treatment.^8^ Therefore, continuous explorations of the relationship between epigenetic mechanisms in the plaque formation and rupture will provide novel insights into the aetiology and potential therapeutic targets.^9^

Coronary artery disease was considered to be caused by a series of inflammatory procedures and promoted by oxidative stress.^10^, ^11^ The imbalance in the oxidant/antioxidant mechanisms caused by excessive reactive oxygen species (ROS) leads to a state of oxidative stress.^12^ TXNIP, one of the α‐arrestin protein superfamily, is originally found as an oxidative stress modulator by inhibiting the activity of thioredoxin.^13^ Depending on the accumulation of ROS and generation of oxidative stress, TXNIP also plays important roles in inflammatory responses^14^ and cell apoptosis.^15^ TXNIP could bind to the NOD‐like receptor family, pyrin domain containing 3 (NLRP3) in response to oxidative stress and activate the NLRP3‐inflammasome to regulate inflammation, which contributes to diabetes and Alzheimer's disease.^16^, ^17^ In diabetes, increased TXNIP has close association with hypertriglyceridemia and high diastolic blood pressure^16^, ^18^ and could induce the apoptosis of pancreatic beta cells.^19^ Besides, TXNIP is a key regulator for glucose homeostasis by interacting with the transcription factor carbohydrate response element‐binding protein (ChREBP).^20^

Various studies showed that TXNIP was overexpressed in peripheral blood leucocytes (PBLs) of CAD patients.^21^, ^22^, ^23^ However, the specific mechanism of it remained unclear. Recent researches revealed that cg19693031, a methylated position at the 3′ UTR of TXNIP, was associated with type 2 diabetes (T2DM).^24^, ^25^ In addition, cg19693031 was correlated with the sustained hyperglycaemia.^26^ Therefore, combined with our previous study,^23^ we hypothesized that overexpressed TXNIP regulated by demethylated cg19693031 might play important role in the inflammatory responses and activation of monocytes, which provided compelling evidences for TXNIP serving as a risk factor for CAD. So, we executed the following study to explore the function of TXNIP in CAD.

## 2 MATERIAL AND METHODS

### 2.1 Clinical cohorts

From December 2017 to February 2019, a total of 259 individuals including 131 CAD patients and 128 sex‐ and age‐matched control subjects (non‐CAD) were recruited in Zhongnan Hospital of Wuhan University (Hubei, China). The inclusion and exclusion criteria in this paper are consistent with previous studies.^27^ Fasting plasma glucose (FPG) and lipid levels (total cholesterol (TC), total triglyceride (TG), high‐density lipoprotein cholesterol (HDL‐C) and low‐density lipoprotein cholesterol (LDL‐C)) were all detected by standard techniques in Core Laboratory of Zhongnan Hospital. Relevant data, including smoking status, histories of hypertension, hyperlipidemia, T2DM and other clinical data of all participants, were collected. This study was approved by the Ethics Committees of Zhongnan Hospital of Wuhan University and followed the Declaration of Helsinki.

### 2.2 DNA extraction and pyrosequencing in peripheral blood leucocytes

Genomic DNA was extracted from PBLs of 108 individuals (54 CAD and 54 controls) from the total samples using phenol/chloroform method and quantified by NanoDrop 2000 (Thermo Scientific). Bisulphite treatment of genomic DNA (1 μg) was conducted using the EZ DNA Methylation Kit (Zymo Research), and bisulphite‐treated DNA (~20 ng) was amplified using the PyroMark PCR kit (Qiagen). Then, methylation levels were quantified by pyrosequencing using the PyroMark Q96 MD instrument (Qiagen). Non‐CpG cytosines, fully methylated and unmethylated DNA were used for quality controls. Methylation level for each sample was calculated as the mean of two independent runs.

### 2.3 Cell culture

The cells in our work were obtained from China Center for Type Culture Collection (CCTCC), and incubated at 37°C in 5% CO_2_. THP‐1 cells were cultured in RPMI 1640 medium (Gibco) supplemented with 10% foetal bovine serum (FBS, Gibco). Human umbilical vein endothelial cells (HUVECs) were cultured with EBM‐2 (Lonza) with endothelial growth media supplements.

### 2.4 Demethylation and bisulphite sequencing in cell lines

THP‐1 cells were seeded into 60‐mm dishes at a density of 2 × 10^6^ cells/mL, and a certain amount of 5‐azacytidine was added into RIPM 1640 medium to a final concentration of 0, 0.5 and 1 μmol/L. Then, cells were cultured in 37°C, and refreshed with the medium in 24 and 48 hours, to maintain the concentration of 5‐azacytidine. After 72 hours, RNA and DNA were extracted. TXNIP mRNA expression level was determined by qPCR. DNA (~20 ng) was bisulphite‐treated as before and then amplified using the EpiTaq™ HS PCR kit (TaKaRa). The PCR products were purified by gel electrophoresis and cloned by a pMD™19 T‐Vector Cloning Kit (Takara). Then, methylation levels at cg19693031 were calculated based on the sequencing results of ten clones in an ABI3730 (Agilent).

### 2.5 Lentivirus transfection

THP‐1 cells were seeded in a 12‐well plate at a density of 4 × 10^5^ cells/mL and then transfected with lentivirus (Genechem). The TXNIP‐expressing lentivirus (TXNIP‐lentivirus group, TXNIP‐lentivirus) or the corresponding control lentivirus (control lentivirus group, CON‐lentivirus) was gently suspended in monocytes by determination of multiplicity of infection (MOI = 80). After incubation at 37°C for 12 ~ 16 hours, the virus venom was removed. TXNIP‐shRNA lentivirus (TXNIP‐shRNA) or control lentivirus (con‐shRNA) was transfected following the same procedure. Transfected cells were further cultured in RMPI 1640 medium for further experiments.

### 2.6 RNA extraction and real‐time quantitative polymerase chain reaction

Total RNA of PBLs and THP‐1 was extracted using TRIzol reagent (Invitrogen) in accordance with the manufacturer's instructions. RNA was qualitied using NanoDrop 2000 and then reversely transcribed into cDNA using Reverse Kit (TOYOBO). Real‐time quantitative polymerase chain reaction (qPCR) was performed using Bio‐Rad CFX 96 real‐time system (Bio‐Rad Laboratories (Shanghai) Co., Ltd.) and quantified by SYBR Prime Script qPCR kit (CWBIO). The relative gene expression of mRNA was calculated using the comparative crossing threshold cycle threshold Ct (2^−ΔΔCt^) method and fold change values. *GAPDH* was used as an internal reference gene. All samples were run in triplicate, and the result was discarded when a difference higher than 0.5 was observed between the duplicates.

### 2.7 Protein extraction and Western blot analysis

Cells were harvested in RIPA lysis buffer (Beyotime) supplemented with 1 μmol/L PMSF (Sigma) and Protease Inhibitor Mixture (Roche Diagnostics). Cell lysates were placed on ice for 30 minutes and centrifuged for 25 minutes (16 000 *g*, 4°C). The concentration of proteins was measured by BCA kit (Beyotime). A total of 25 μg proteins was separated by SDS‐polyacrylamide gel electrophoresis (SDS‐PAGE), transferred to PVDF membranes and subsequently blocked in 5% bovine serum albumin (BSA) for 2 hours. Then, the membranes were incubated overnight at 4°C with appropriate diluted primary antibodies as following: anti‐TXNIP (1:1000), anti‐GAPDH (1:50 000). Secondary antibodies (1:10 000) were incubated at room temperature for 1 hour after being washed for 4 times (10 minutes per time) with 1% TBST (0.1% Tween‐20). Images were conducted by Tanon‐5200 Chemiluminescent Imaging System (Tanon Science & Technology). Densitometric analyses of membranes were performed using ImageJ software (version 2.0).

### 2.8 Adhesion assay

Human umbilical vein endothelial cells were seeded into 12‐well plates at a density of 3 × 10^5^ cells/mL and cultured in 37°C for 1 day prior to assays, and the confluence of cells was evaluated under the microscope. THP‐1 cells (5 × 10^5^ cells/mL) stained with 5 μmol/L BCECF‐AM (Beyotime) in the dark for 30 minutes were added onto confluent monolayers of HUVECs and co‐cultured at 37°C for 60 minutes. Non‐adherent cells were removed and washed with PBS for twice. Then, attached cells were observed with an inverted fluorescence microscope and photographed in five random fields per well at 100× magnification. The number of adherent cells was quantified using the ImageJ software. This assay was performed in triplicate.

### 2.9 Transwell assay

The migration assay was performed using a Transwell insert plates with an aperture of 8 µm (Corning Incorporated) in 24‐well plates. Monocyte chemoattractant protein‐1 (MCP‐1; Beyotime) was dissolved in PBS that contained 0.1% bovine serum albumin (BSA). About 5 × 10^5^ of THP‐1 cells were placed in the upper chamber with RPMI 1640 medium, while RPMI 1640 medium supplemented with 5% FBS and 200 ng/mL MCP‐1 was placed in the lower chamber. After cells were incubated for 90 minutes at 37°C, the culture medium was removed and cells were washed with PBS twice. The cells on the upper side of filters were removed using cotton‐tipped swabs, and the cells on the underside of filters were fixed in 4% paraformaldehyde (biosharp) for 10 minutes and stained with 0.1% crystal violet staining solution (Sinopharm) for 10 minutes at room temperature. The migrated cells were counted in five random fields for each well at 100× magnification under the inverted microscope. The migration assay was duplicated in triplicate.

### 2.10 TXINP expression and methylation changes in the formation of THP‐1 derived macrophages and foam cells

In vitro the classic cell model was conducted on THP‐1 for exploring the roles of TXNIP in the formation of macrophages and foam cells. Concretely, THP‐1 cells were induced into macrophages by treating with 75 ng/mL PMA for 24 hours and partial cells were collected to extract DNA and RNA. Then, residual cells were cultured in the medium supplemented with 50 mg/L ox‐LDL to be induced into foam cells. Some were used for oil red O staining, while others were collected at 12 and 24 hours to extract DNA and RNA, which were used to determine the methylation of cg19693031 and the expression of TXNIP, respectively.

### 2.11 Statistical analysis

The data were expressed as the mean ± standard deviation (SD), median (inter‐quartile range) or number of cases (percentage). The differences in quantitative between cases and controls were assessed by the two‐tailed Student's *t* test (for normal distributed data) or non‐parametric test (Mann‐Whitney *U* test, for skewed data), while unordered categorical variables were compared by chi‐squared (χ^2^) test, respectively. The Pearson (for normal distributed data) or Spearman (for skewed data) tests were applied to confirm the correlations of variables. All analyses were performed with GraphPad Prism 6.0 (GraphPad Software) and SPSS 19.0 software (SPSS, Inc). A *P* < .05 was considered to be statistically significant.

## 3 RESULTS

### 3.1 Clinical characteristics

Characteristics of all participates are shown in Table 1. Control individuals (CON) are age‐ and sex‐matched with CAD patients (CAD) (*P* > .05). Compared with CON, the percentage of neutrophils and monocytes was significantly increased in CAD. On the contrary, the percentage of lymphocytes was significantly decreased in CAD. CAD patients had higher distributions of diabetes, hypertension, smoking and hyperlipidemia (*P* < .05). Levels of FPG, TG and LDL‐C in CAD were higher than CON, while HDL‐C levels in CAD were lower than CON (*P* < .05). However, no differences in TC levels and PBLs amounts were detected between two groups.

**Table 1:** Clinical characteristics of the CAD and control (non‐CAD) individuals

Clinical data	Control (n = 127)	CAD(n = 131)†	*P*‐value
Age (y)	61 (58‐67)	63 (58‐70)	.275^b^
Male, gender	68 (53.5)	75 (57.3)	.549^c^
WBC	5.69 (5.11‐6.48)	5.83 (5.13‐7.3)	.197^b^
NEUT%****	*56.32 ± 6.61*	*63.54 ± 10.30*	<*.0001* a
LYMPH%****	*33.52 ± 6.09*	*25.81 ± 8.10*	<*.0001* a
MONO%*	*7.1 (6.25‐8.10)*	*7.56 (6.40‐9.00)*	*.025* b
TC (mmol/L)	4.6 (4.15‐4.90)	4.38 (3.47‐5.02)	.109^b^
TG**** (mmol/L)	*1.01 (0.81‐1.25)*	*1.35 (0.94‐1.98)*	<*.0001* b
HDL‐C**** (mmol/L)	*1.34 (1.16‐1.64)*	*1.07 (0.92‐1.20)*	<*.0001* b
LDL‐C** (mmol/L)	*2.63 ± 0.51*	*2.79 ± 0.84*	*.007* a
Diabetes****, n (%)	*1 (0.8)*	*49 (37.4)*	<*.0001* c
Hypertension****, n (%)	*23 (18.1)*	*82 (62.6)*	<*.0001* c
Smoking**, n (%)	*26 (20.5)*	*49 (37.4)*	*.003* c
Hyperlipidemia****, n (%)	*3 (2.4)*	*26 (19.8)*	<*.0001* c
FPG (mmol/L)****	*5.31 (4.97‐5.60)*	*5.75 (5.24‐6.85)*	<*.0001* b
TXNIP mRNA level*	*0.911 (0.621‐1.230)*	*0.976 (0.726‐1.290)*	*.041* b

Note

The results are presented as mean ± SD (standard deviation) or as median (inter‐quartile range); Italic letters show the significant associations and their *P*‐values.

Abbreviations: FPG, fasting plasma glucose; HDL‐C, high‐density lipoprotein cholesterol; LDL‐C, low‐density lipoprotein cholesterol; LYMPH, lymphocyte; MONO, monocyte; NEUT, neutrophil; TC, total cholesterol; TG, triglyceride; WBC, white blood cell.

a Two‐tailed Student's *t *test.

b Non‐parametric test (Mann‐Whitney *U *test).

c χ^2^ test.

^†^ The CAD patients are consisted of 40 (30.5%) myocardial infarction, 34 (26%) stable angina pectoris, and 57 (43.5%) unstable angina pectoris, respectively.

*P < .05, **P < .01, ***P < .001 and ****P < .0001.

### 3.2 Decreased methylation of cg19693031 in the 3′ UTR of TXNIP and was correlated with elevated TXNIP mRNA level

**Figure 1:**
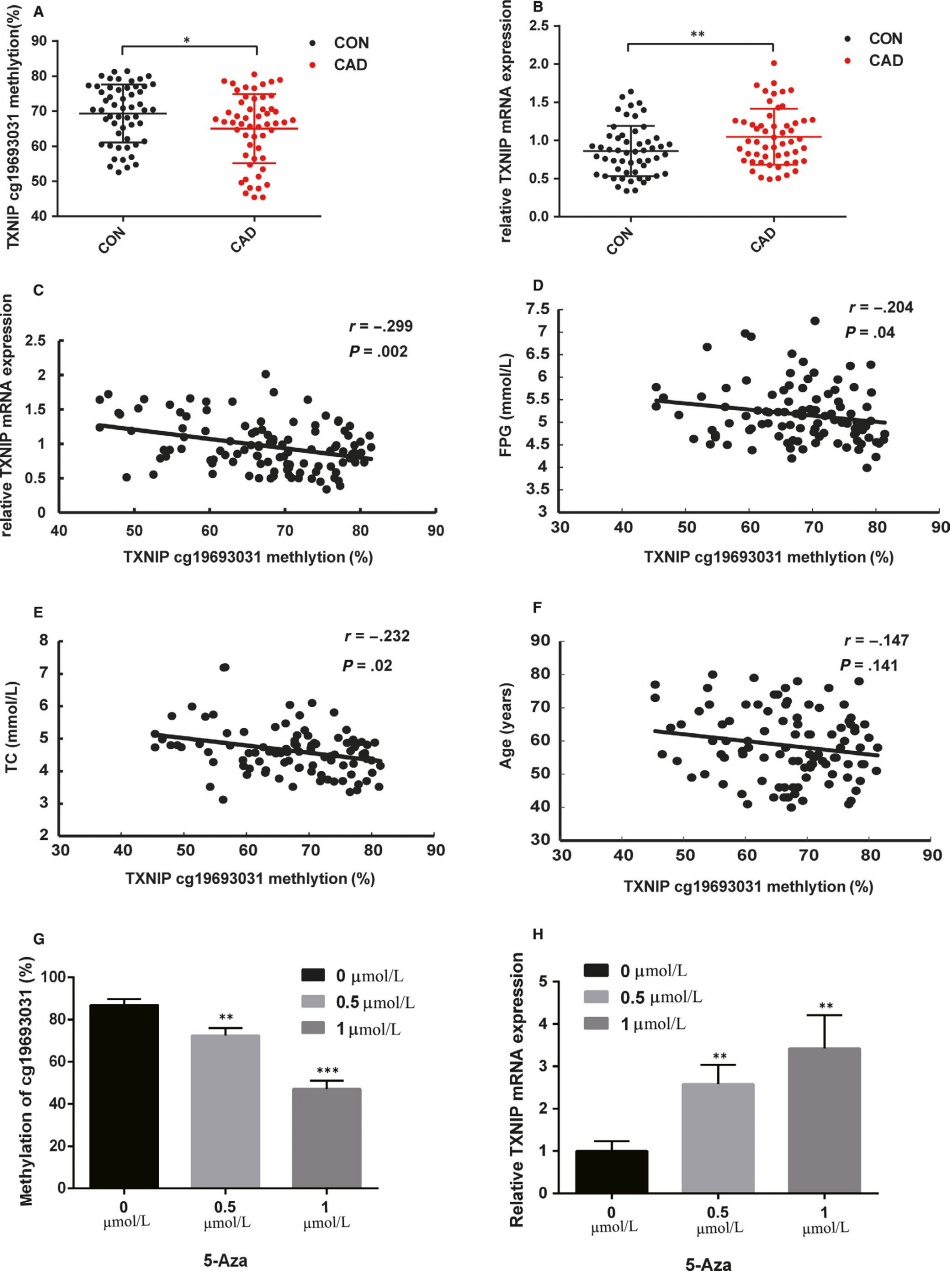
Negative correlation with methylation at cg19693031 and mRNA expression of TXNIP in PBLs and 5‐Aza treated THP‐1 cells. A, The DNA methylation levels of cg19693031 (in the 3′ UTR of TXNIP) were detected by pyrosequencing in the PBLs of CAD patients (n = 54) and controls (n = 54). B, The mRNA levels of TXNIP were detected by qPCR in the PBLs of CAD and controls. C‐F, The correlations of DNA methylation at cg19693031 and mRNA level of TXNIP (C), the level of TC (D), the level of GLU (E) in the plasma and the age (F) of all participates. G, The DNA methylation levels of cg19693031 were detected by bisulphite sequencing in THP‐1 treated with 5‐Aza at different concentrations. H, The mRNA levels of TXNIP were detected by qPCR in THP‐1 treated with 5‐Aza at different concentrations. All values are the averages of three independent experiments, and the results are present as the means ± SDs. *P* < .05 was defined as significant. *P < .05; **P < .01. *r*: Spearman correlation coefficient; 5‐Aza: 5‐azacytidine

DNA isolated from PBLs of 54 CAD and 54 control individuals were used to investigate the methylation of cg19693031 using pyrosequencing. The clinical information of these individuals was shown in Table 2. The methylation of cg19693031 in CAD was significantly decreased compared with controls (Figure 1A,B) and negatively correlated with TXNIP expression levels (*r* = −.299, *P* = .002) (Figure 1C). Similar associations were also seen between the TXNIP expression and FPG or TC levels (*r*_FPG_ = −.204, *P* = .04; *r*_TC_ = −.232, *P* = .02) (Figure 1D,E). However, we did not observe significant correlation between age and the methylation of cg19693031 (*r*_Age_ = −.147, *P* = .141) (Figure 1F). The methylation of cg19693031 and mRNA expression of TXNIP in three subtypes of CAD were shown in Figure S1.

**Table 2:** Clinical characteristics of the CAD and control (non‐CAD) individuals involved in pyrosequencing

Characteristics	Control (no‐CAD, n = 54)	CAD(n = 54)	*P*‐value
Age (y)	61 (54.75‐65)	62.5 (56.75‐70.25)	.166^a^
Male, n (%)	32 (59.3)	29 (53.7)	.56^c^
Systolic blood pressure	128 (117.25‐142)	137 (118.5‐153.75)	.125^a^
Diastolic blood pressure	79 (71.25‐88)	80 (73‐89.75)	.33^a^
FPG* (mmol/L)	*4.97 (4.75‐5.27)*	*5.25 (4.53‐5.61)*	*.039* a
TC**** (mmol/L)	*4.33 (3.90‐4.72)*	*4.8 (4.51‐5.44)*	<*.0001* a
TG* (mmol/L)	*1.14 (0.93‐1.36)*	*1.3 (0.96‐2.19)*	*.01* a
HDL‐C**** (mmol/L)	*1.31 (1.12‐1.52)*	*1.02 (0.96‐1.12)*	<*.0001* a
LDL‐C** (mmol/L)	*2.65 (2.32‐3.04)*	*3.01 (2.86‐3.25)*	*.001* a
Diabetes**, n (%)	*4 (7.4)*	*15 (27.8)*	*.005* c
Hypertension****, n (%)	*9 (16.7)*	*31 (57.4)*	<*.0001* c
Smoking*, n (%)	*12 (22.2)*	*22 (40.7)*	*.031* c
cg19693031 methylation* (%)	*70.4 (61.9‐76.8)*	*66.78 (57.2‐72.75)*	*.023* a
TXNIP mRNA level*	*0.90 ± 0.34*	*1.05 ± 0.37*	*.027* b

Note

The results are presented as mean ± SD (standard deviation) or as median (inter‐quartile range); Italic letters show the significant associations and their *P*‐values.

Abbreviations: FPG, fasting plasma glucose; HDL‐C, high‐density lipoprotein cholesterol; LDL‐C, low‐density lipoprotein cholesterol; TC, total cholesterol; TG, triglyceride.

a Two‐tailed Student's *t* test.

b Non‐parametric test (Mann‐Whitney *U* test).

c χ^2^ test.

*P < .05, **P < .01, ***P < .001 and ****P < .0001.

To further verify the correlation between TXNIP expression and methylation in vitro, we used different concentrations of 5‐azacytidine to induce THP‐1 cells. Notably, the results of the bisulphite sequencing and qPCR showed that decreased methylation level of cg19693031 and increased TXNIP mRNA expression were found in the THP‐1 cells treated with 5‐azacytidine; moreover, there were statistically significant differences among groups treated with different dosages of 5‐azacytidine (Figure 1G,H).

### 3.3 Elevated expression of TXNIP and inflammatory markers in PBLs of patients

**Figure 2:**
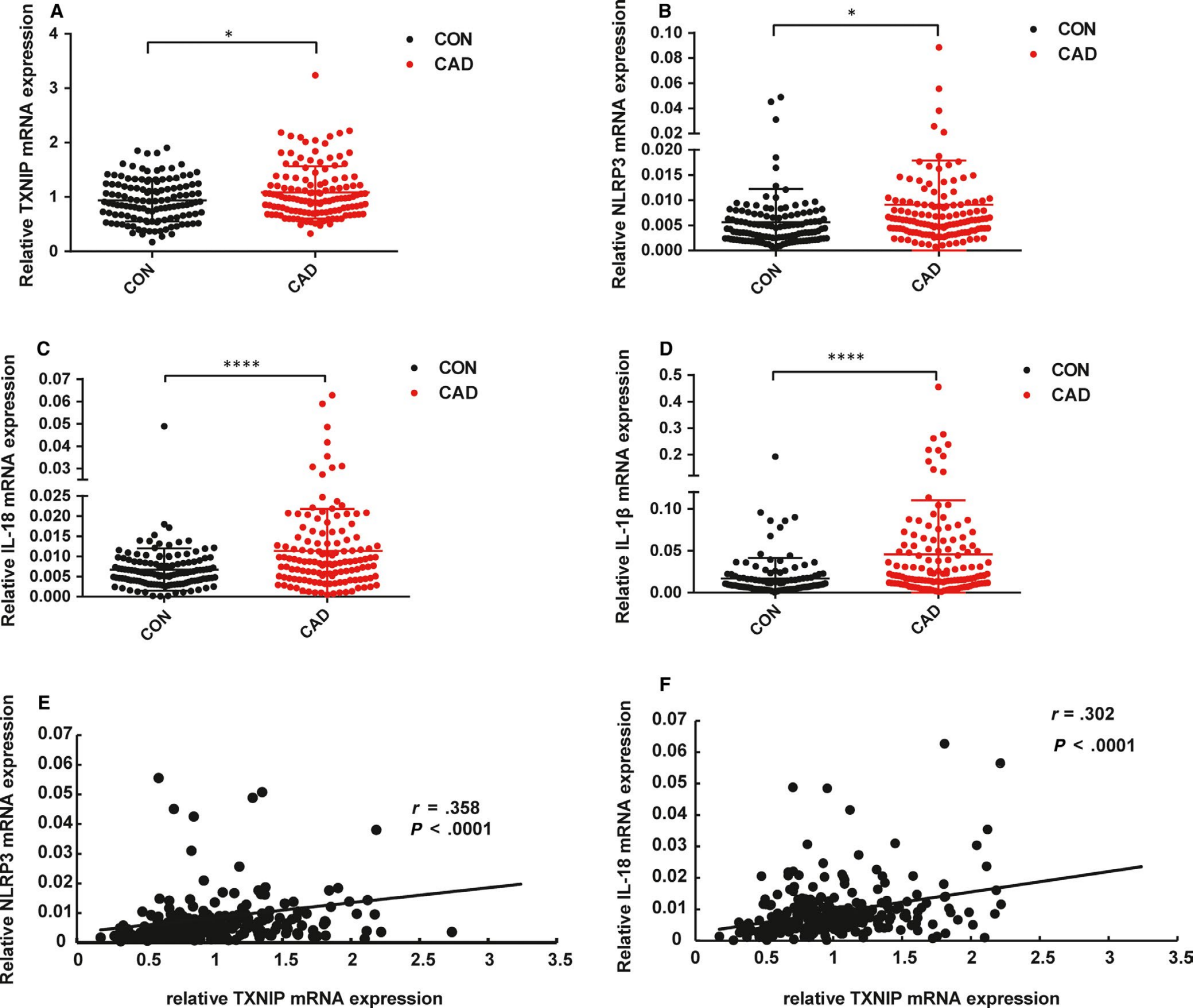
The mRNA expression of TXNIP, NLRP3, IL‐1β and IL‐18 in PBLs of CAD (n = 131) and control (n = 128) individuals. A‐D, The mRNA expression of TXNIP (A), NLRP3 (B), IL‐1β (C) and IL‐18 (D) was detected by qPCR in PBLs of CAD and control individuals. E, Associations of TXNIP and NLRP3 in all of two groups. F, Associations of TXNIP and IL‐18 in all of two groups. All values are the average of at least three replicates, and the results are present as the means ± SDs. *P* < .05 was defined as significant. *P < .05; **P < .01; ***P < .001; ****P < .0001. *r*: Spearman correlation coefficient

The expression of TXNIP in CAD was significantly increased compared with CON (*P* < .05) (Figure 2). Besides, NLRP3, IL‐1β and IL‐18 mRNA expression were significantly increased in CAD. Moreover, the expression of NLRP3 and IL‐18 was positively correlated with TXNIP expression (*r*_NLRP3_ = .358, *P* < .0001; *r*_IL‐18_ = .302, *P* < .0001). However, there was no statistical positive correlation between TXNIP and IL‐1β (The result was not shown). The detailed information of qPCR primers was listed in Table S1.

### 3.4 TXNIP overexpression promotes the expression of inflammatory markers and activation of monocytes

**Figure 3:**
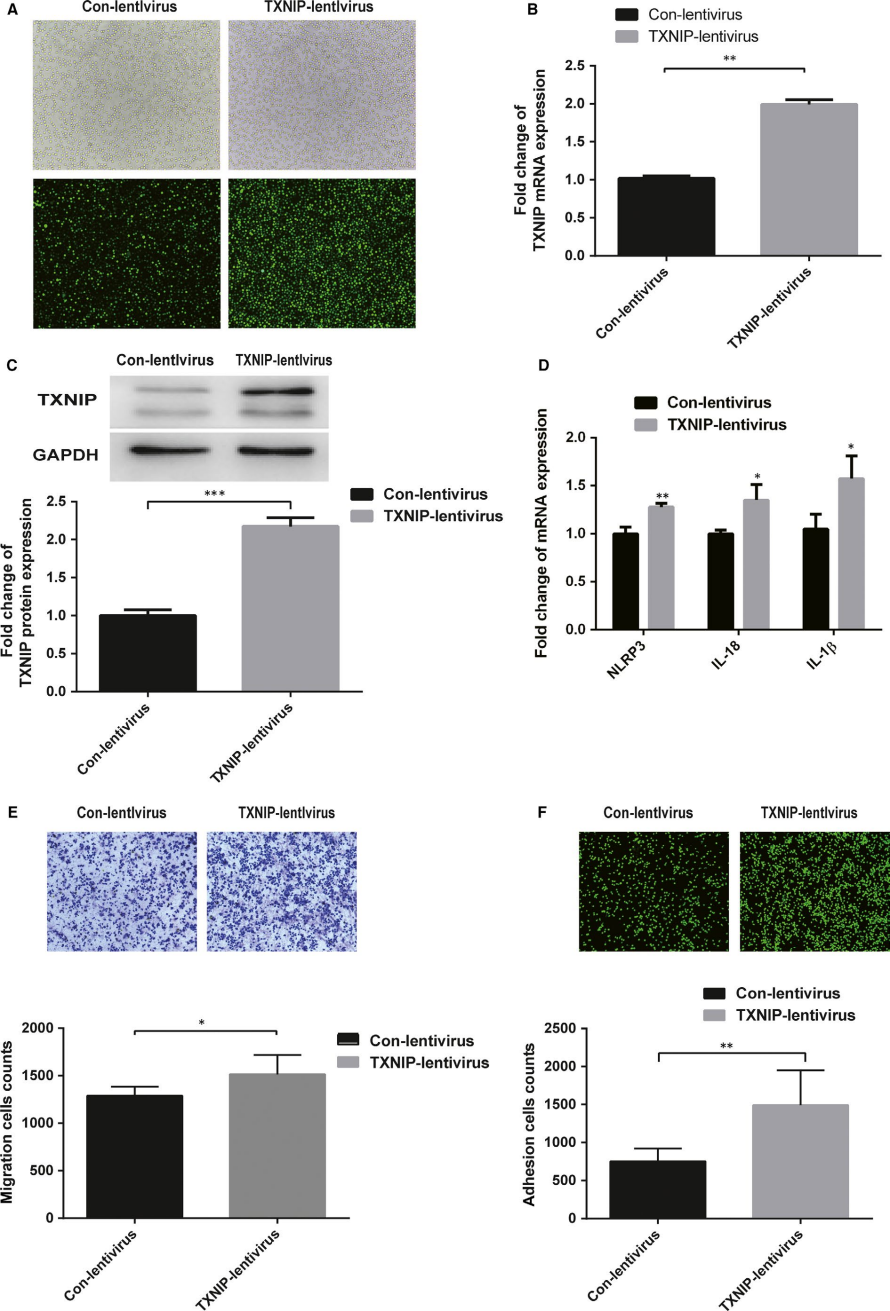
Overexpression of TXNIP increased TXNIP expression and promoted monocyte activation. A, Fluorescent and white light images of THP‐1 cells after infected with TXNIP‐lentivirus or CON‐lentivirus. B and C, The relative TXNIP mRNA (B) and protein (C) expression was determined by RT‐qPCR and Western blotting in THP‐1 cells transfected with TXNIP‐lentivirus or with CON‐lentivirus in three independent experiments. D, The relative mRNA expression of NLRP3, IL‐18 and 1β was determined by RT‐qPCR in three independent experiments. E, The migration capacity of THP‐1 cells in three groups was assessed using a fluorescence microscope (100×). F, The adhesive capacity of THP‐1 cells in three independent experiments was assessed by Transwell migration assay with an inverted microscope (100×). Results are the means ± SDs for three independent experiments. *P* < .05 was defined as significant. *P < .05; **P < .01

To investigate whether TXNIP is involved in regulating the inflammatory response and the activation of monocytes, THP‐1 cells were transfected with the TXNIP lentiviral expression vector expressing green fluorescent (GFP) protein (TXNIP‐lentivirus) and the empty lentiviral vector expressing GFP protein (CON‐lentivirus). After 72 hours, the efficiency of virus infection was determined by observing the green fluorescence with an inverted fluorescence microscope at 300× magnification (Figure 3A) and then verified by TXNIP mRNA (Figure 3B) and protein expressions (Figure 3C).

Compared with the control group, TXNIP‐lentivirus transfected cells showed increased mRNA expression of NLRP3, IL‐18 and IL‐1β (Figure 3D), and the numbers of adherent and migrated THP‐1 cells were significantly increased (Figure 3E).

### 3.5 TXNIP knock‐down inhibits the expression of inflammatory markers and activation of monocytes

**Figure 4:**
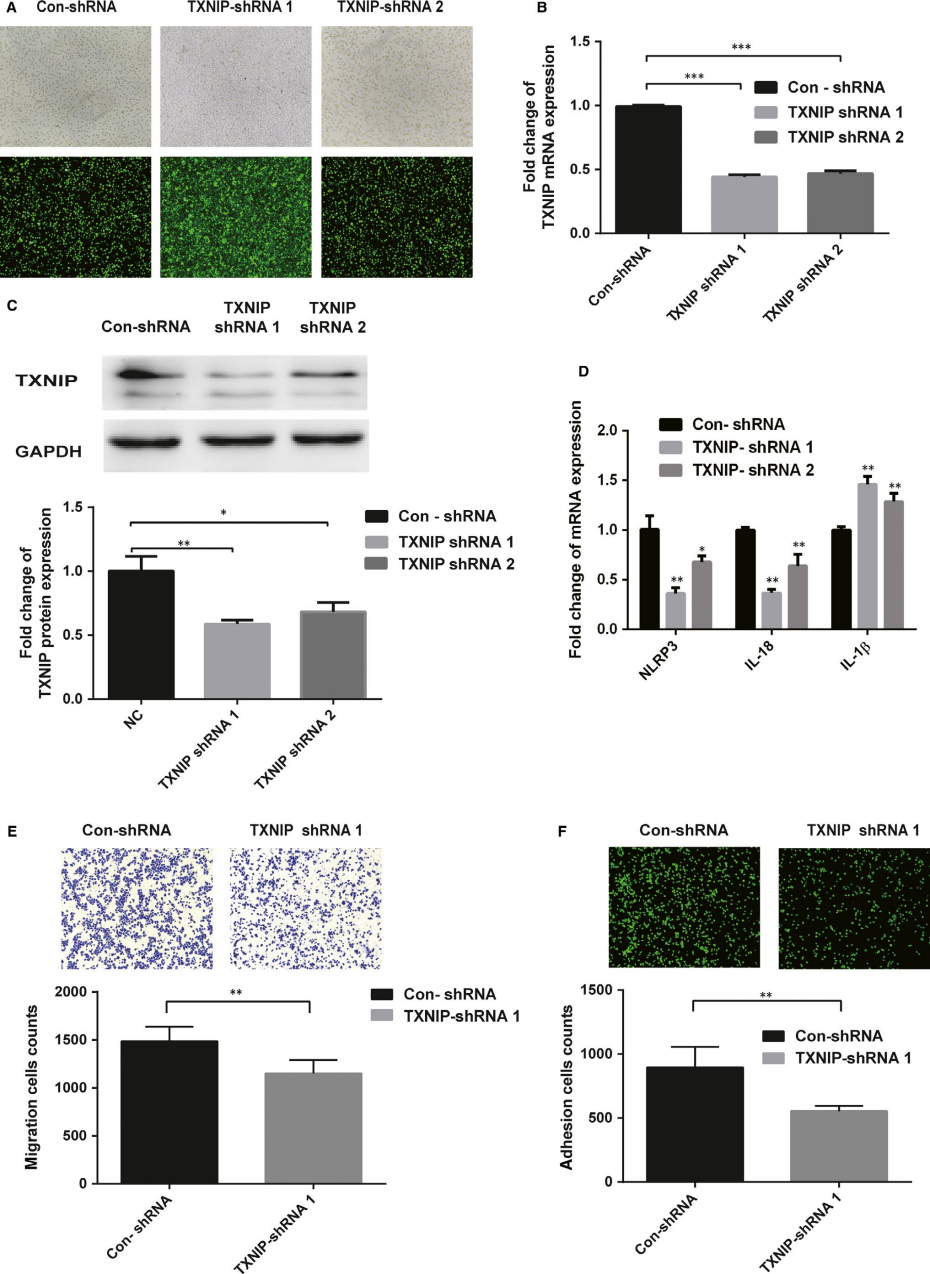
Knock‐down of TXNIP decreased TXNIP expression and inhibited monocyte activation. A, Fluorescent and white light images of THP‐1 cells after infected with TXNIP‐shRNA1, TXNIP‐shRNA2 or CON‐shRNA. B and C, The relative TXNIP mRNA (B) and protein (C) expression was determined by RT‐qPCR and Western blotting in THP‐1 cells transfected with TXNIP‐shRNA1, TXNIP‐shRNA2 or with CON‐shRNA in three independent experiments. D, The relative mRNA expression of NLRP3, IL‐18 and 1β was determined by RT‐qPCR in three independent experiments. E, The migration capacity of THP‐1 cells in three groups was assessed using a fluorescence microscope (100×). F, The adhesive capacity of THP‐1 cells in three groups was assessed by Transwell migration assay with an inverted microscope (100×). Results are the means ± SDs for three independent experiments. *P* < .05 was defined as significant. *P < .05; **P < .01

THP‐1 was transfected with two kinds of TXNIP‐shRNA lentivirus vectors (TXNIP‐shRNA 1 and TXNIP‐shRNA 2), and a control‐shRNA lentivirus vector (con‐shRNA), which induced the decreased expression of NLRP3, IL‐18 and IL‐1β in the TXNIP‐shRNA groups. The sequences of TXNIP‐shRNA and control‐shRNA were shown in Table S2. However, considering the efficiency of TXNIP knock‐down, we chose TXNIP‐shRNA 1 to further perform the adhesion and migration assay. As shown in Figure 4, TXNIP knock‐down inhibited the adhesion and migration ability of THP‐1.

### 3.6 Elevated TXNIP and decreased methylation at cg19693031 in the THP‐1 derived macrophages and foam cells

Macrophages and foam cells are particularly important in atherosclerotic plaque formation. Compared with THP‐1 cells, we found THP‐1‐derived macrophages and foam cells phagocytized many orange‐red lipid particles, have a continuously increased TXNIP expression, while the methylation at cg19693031 was decreased (Figure S2).

## 4 DISCUSSION

Our study revealed that TXNIP expression was regulated by the methylation of cg19693031 and positively related to NLRP3, IL‐18 expressions in CAD. Moreover, cytological experiments confirmed that TXNIP could promote the migration and adhesion of monocytes. Thus, we considered that elevated TXNIP induced by demethylated cg19693031 might activate NLRP3 inflammasome, triggering a series of subsequent inflammatory responses and promoting the activation and inflammation of monocytes and formation of macrophages/foam cells, and ultimately lead to the occurrence of cardiovascular event.

Inflammation induced by oxidative stress persists throughout the formation of AS and results in the occurrence of CAD.^8^, ^28^ TXNIP, as a crucial redox regulator, plays important roles in the process of inflammation, such as the activation of NLRP3 inflammasome and maturation of IL‐1β.^29^ Our results indicated that the mRNA expression of NLRP3, IL‐1β and IL‐18 was up‐regulated and positively correlated with the increased TXNIP in the PBLs of CAD and THP‐1 cells, which is consistent with that TXNIP could promote vascular inflammatory responses and accelerate the process of AS by activating NLRP3 inflammasome.^30^, ^31^, ^32^

Circulating immune cells have attracted many attentions and involved in CAD.^33^, ^34^ Previous reports revealed ratios of mononuclear and neutrophil were increased in CAD and maybe independent risks.^35^ Besides, monocytes dysfunction is equally important in the progression of CAD.^36^, ^37^ A series of processes such as adhesion, migration of monocytes and formation of phagocytic lipid macrophages are the key trigger factors of atherosclerotic plaques.^38^ It was reported that TXNIP could be up‐regulated in the condition of disturbed flow and involved in promoting endothelial cells dysfunction in CAD.^32^, ^39^ Moreover, our results revealed TXNIP could activate monocytes to promote the progression of AS in all stages.

Site‐specific DNA methylation could regulate gene expression and lead to AS. For example, dysregulated cystathionine γ‐lyase induced by DNA methylation could contribute to the monocyte‐induced inflammation and AS.^40^ Hypomethylation of long interspersed nuclear element‐1 was relevant to myocardial infarction risk.^41^ In this work, we found that methylation of cg19693031 was decreased in the PBLs of CAD and negatively associated with increased TXNIP expression, which was further verified in THP‐1 cells. These indicated that cg19693031 might act as a vital role in the pathomechanism of CAD via regulating TXNIP expression.

Researchers have committed to investigating the upstream regulators on the adjustment of DNA methylation in recent years, but no report has demonstrated the upstream mechanism of cg19693031. In our study, TC and FPG levels were both significantly relevant to methylation of cg19693031, which was coincident to the previous study.^25^, ^26^, ^42^, ^43^ Specifically, studies suggested that DNA methylation could be altered by dyslipidemia and the stimulation of insulin or glucose.^44^, ^45^ For example, insulin could lead to changed DNA methylation in the 3′ UTR of ATP2A3. Dysregulated lipid metabolism could induce overexpressed TXNIP by promoting endoplasmic reticulum (ER) stress.^16^ At the same time, ER stress could induce MCP‐1 overexpression by SET7/9‐mediated histone methylation.^46^ Therefore, we speculated that glucolipid metabolic might involve in the regulation of the methylation level at cg19693031. On the other hand, the methylation of cg19693031 in PBLs might reflect the methylation status in pancreas, which could be the cause of the insulin resistant and further hyperglycaemia and dyslipidemia in diabetes.^19^, ^20^

Moreover, studies suggested microRNAs were involved in the regulation of DNA methylation.^47^ For example, miR‐126, miR‐148a and miR‐21 could regulate methylation of CD4^+^ T cells and contribute to systemic lupus erythematosus.^48^, ^49^ Thus, there also might be potential microRNAs involved in the modulation of cg19693031.

Nevertheless, our study still has some limitations. For one thing, the sample size is small, so there may be some confounding factors influencing our results. Additionally, without isolating monocytes from PBLs of healthy individuals, we only performed cytological experiments in THP‐1 cells. Finally, this study is lack of the verification of animal experiment. Thus, further investigation is required to explain the function of TXNIP in monocytes.

## 5 CONCLUSION

In conclusion, we proposed that the up‐regulated TXNIP influenced by the demethylation of cg19693031 in CAD patients orientated monocytes towards an inflammatory activated state, probably through the NLRP3 inflammasome pathway and played important roles in the formation of macrophage and foam cells. All of these suggested a potential epigenetic and inflammatory pathway in monocytes regulated by TXNIP involved in the pathogenesis of CAD and revealed TXNIP may be a novel therapeutic target in CAD.

## 6 AUTHOR’ CONTRIBUTIONS

JLR, FZ and XXQ designed the study. JLR conducted all experiments and statistical analysis. XKZ and YX took part in cohort study. GHY, BL and SYH participated in the cell experiments. JLR, FZ and XLM finalized the manuscript. All authors read and approved the final manuscript.

## CONFLICT OF INTEREST

The authors confirm that there are no conflicts of interest.

## Data Availability

The data that support the findings of this study are available on request from the corresponding author. The data are not publicly available due to privacy or ethical restrictions.
