# Supplementary figures and images for: Thioredoxin‐interacting protein promotes activation and inflammation of monocytes with DNA demethylation in coronary artery disease
Source: J Cell Mol Med. 2020 Feb 10;24(6):3560–71. doi: 10.1111/jcmm.15045 (PMC7131938; doi:10.1111/jcmm.15045)

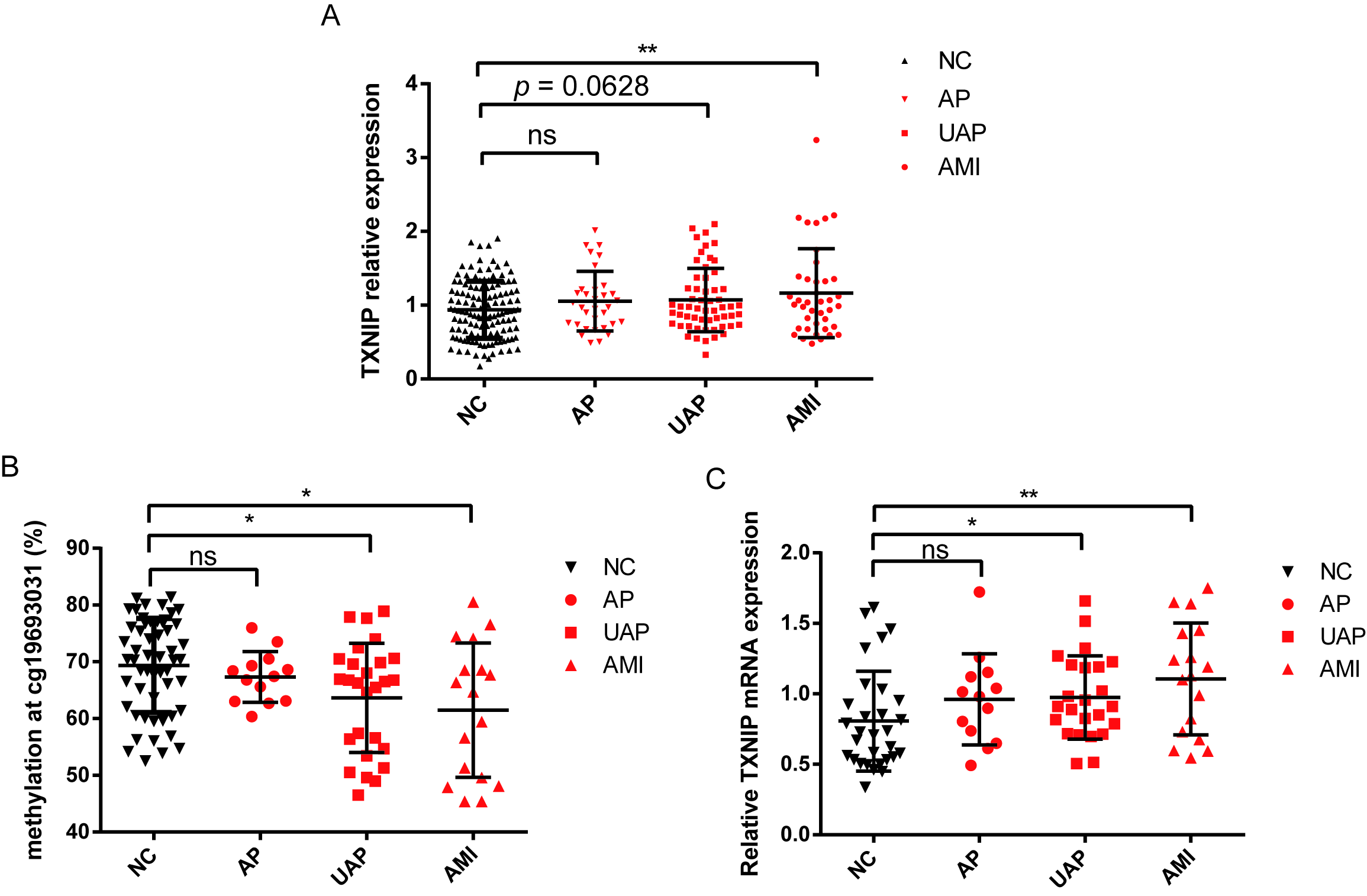

Supplement: Supplementary file 1 [file JCMM-24-3560-s001.tif]

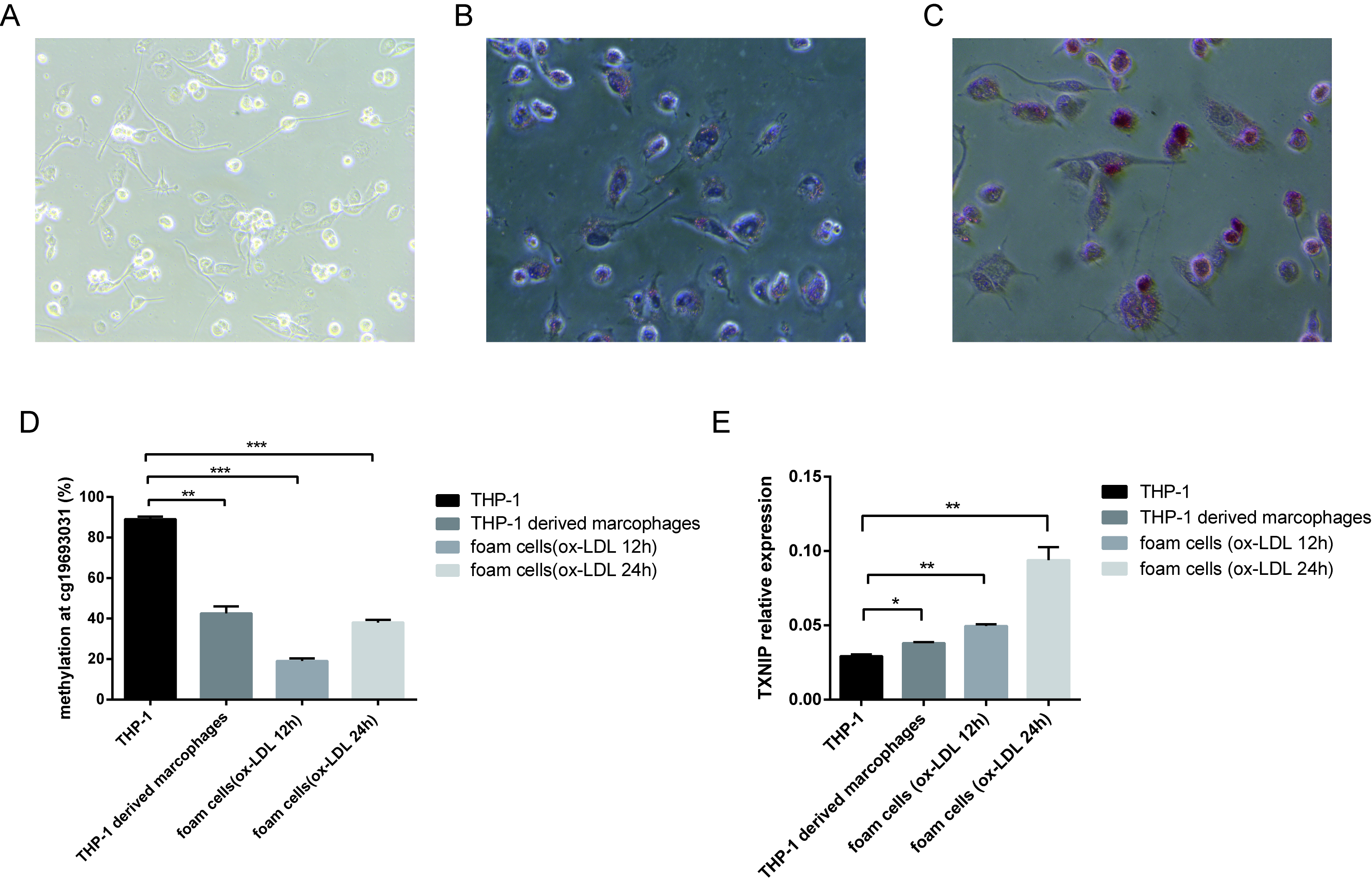

Supplement: Supplementary file 2 [file JCMM-24-3560-s002.tif]
