# Supplementary material for: Thioredoxin‐interacting protein promotes activation and inflammation of monocytes with DNA demethylation in coronary artery disease
Source: J Cell Mol Med. 2020 Feb 10;24(6):3560–71. doi: 10.1111/jcmm.15045 (PMC7131938; doi:10.1111/jcmm.15045)
Supplement: Supplementary file 3 [file JCMM-24-3560-s003.doc]

**Table S1. The detailed information of RT-qPCR and Pyrosequencing primers.**

| Method | Variable | Primers | Length(bp) | Ann. Temp(°C) |
| --- | --- | --- | --- | --- |
| qPCR | TXNIP | Forward: GCAATCATATTATCTCAGGGAC | 133 | 58 |
|  |  | Reverse: GGAACGCTAACATAGATCAGTAA |  |  |
|  | NLRP3 | Forward: GAGGAAAAGGAAGGCCGACA | 115 | 61 |
|  |  | Reverse: CCCGGCAAAAACTGGAAGTG |  |  |
|  | IL-18 | Forward: TGGCTGCTGAACCAGTAGAAG | 192 | 61 |
|  |  | Reverse: GAGGCCGATTTCCTTGGTCA |  |  |
|  | IL-1β | Forward: TGAGCTCGCCAGTGAAATGA | 143 | 61 |
|  |  | Reverse: AGATTCGTAGCTGGATGCCG |  |  |
|  | GAPDH | Forward: TGTTGCCATCAATGACCCCTT | 202 | 58 |
|  |  | Reverse: CTCCACGACGTACTCAGCG |  |  |
| Pyrosequencing | cg19693031 | #Forward: GTTTGTTGGATGGGTTTAAAAATAATTAGA | 188 | 56 |
|  |  | #Reverse: ACATAAATTCAAAATCCAAAATTCCTATC | |  |
|  |  | Sequencing: GGGTTAGGTAAAAATGG |  |  |

#: The primers were applied in bisulfite genomic sequencing
