# Supplementary material for: Thioredoxin‐interacting protein promotes activation and inflammation of monocytes with DNA demethylation in coronary artery disease
Source: J Cell Mol Med. 2020 Feb 10;24(6):3560–71. doi: 10.1111/jcmm.15045 (PMC7131938; doi:10.1111/jcmm.15045)
Supplement: Supplementary file 4 [file JCMM-24-3560-s004.doc]

**Table.** **S2** **The sequence of TXNIP-shRNA and control.**

| **shRNA** | **Target sequence** |
| --- | --- |
| TXNIP-shRNA 1 | CAGGTCTAAGCAGCAGAACAT |
| TXNIP-shRNA 2 | ATCCATGCTGACTTTGAGAAT |
| TXNIP-CON | TTCTCCGAACGTGTCACGT |
